# Alcohol – a scoping review for Nordic Nutrition Recommendations 2023

**DOI:** 10.29219/fnr.v68.10540

**Published:** 2024-04-01

**Authors:** Dag Steinar Thelle, Morten Grønbæk

**Affiliations:** 1 Department of Biostatistics, Institute of Basic Medical Sciences, University of Oslo, Oslo, Norway; 2 National Institute of Public Health, Copenhagen, Denmark

**Keywords:** Alcohol, total mortality, cause-specific mortality, age, observational studies, Mendelian randomization, safe limits

## Abstract

The objective of this scoping review is to evaluate the updated evidence on the consumption of alcohol and health outcomes regarded as relevant for the Nordic and Baltic countries, including cardiovascular disease, cancer, and all-cause mortality. It is based on the previous Nordic Nutrition Recommendations of 2012 and relevant papers published until 31 May 2021. Current evidence from mainly observational epidemiological studies suggests that regular, moderate alcohol consumption may confer protective effects against myocardial infarction (MI) and type 2 diabetes. Mendelian randomization analyses do not fully support these findings, possibly because these analyses may fail to identify low alcohol intake. For several cancers, it is not possible to set any safe limit. All-cause mortality is not increased with light to moderate alcohol intake in middle-aged and older adults who do not engage in binge drinking. Total abstinence is associated with the lowest risk of mortality in young adults. Observational studies on alcohol consumption are hampered by a number of inherent methodological issues such as ascertainment of alcohol intake, selection of appropriate exposure groups, and insufficient control of confounding variables, colliders, and mediators. It should also be emphasized that there is a socio-economic contribution to the alcohol-health axis with a stronger detrimental effect of alcohol in the lower social classes. The above issues contribute to the complexity of unravelling the causal web between alcohol, mediators, confounders, and health outcome.

## Popular scientific summary

- In the Nordic countries, mean alcohol consumption accounts for 2 to 6% of the total energy intake in adults.
- The intake is unevenly distributed, and some population segments have hazardous intakes.
- Alcohol is a toxic substance that can harm various organs in the body.
- Moderate consumption of 1–2 units a day has been associated with a lower risk of myocardial infarction and type 2 diabetes in observational studies. Mendelian randomization analyses do not fully support these findings, possibly because these analyses may fail to identify low alcohol intake.
- For several cancers, no safe lower limit has been established.
- Binge drinking should be avoided in all age groups.

The aim of this scoping review is to describe the current evidence for selected health-related outcomes as a basis for setting and updating national dietary reference values (DRVs) and food-based dietary guidelines (FBDGs) for alcohol consumption in the Nordic Nutrition Recommendations (NNR) 2023 (Box 1).

*Box 1.* Background papers for Nordic Nutrition Recommendations 2023

- This paper is one of many scoping reviews commissioned as part of the Nordic Nutrition Recommendations 2023 (NNR2023) project (2)
- The papers are included in the extended NNR2023 report but, for transparency, these scoping reviews are also published in Food & Nutrition Research
- The scoping reviews have been peer reviewed by independent experts in the research field according to the standard procedures of the journal
- The scoping reviews have also been subjected to public consultations (see report to be published by the NNR2023 project)
- The NNR2023 committee has served as the editorial board
- While these papers are a main fundament, the NNR2023 committee has the sole responsibility for setting DRVs in the NNR2023 project

Most of the current data on the effect of alcoholic beverages are based on observational studies. These studies are by definition non-experimental, which entails that the researcher does not have full control of all factors that may affect the outcome. The caveats of observational studies comprise classification and ascertainment of exposure as most studies are based on self-reported alcohol and selection of reference groups.

Uncertainty and doubts regarding the effect of potential causal factors are usually good reasons for organizing randomized trials, and especially so if the validity of an observational study may be doubtful. Thus, the ideal data set for alcohol and disease endpoints would have been systematic reviews and meta-analyses based on randomized controlled trials. Such trials are lacking, and one has to make do with well-designed cohort or case-control studies from more than one center or group.

The external validity or generalizability of these studies can only be documented when more than one study confirms the conclusion. This, however, does not solve the major problems with observational studies; how to ensure that the effects of confounding variables, known or unknown, are nullified, including how the researchers tackle the potential influence of previous drinkers in the nondrinker categories.

The instrumental variable approach using Mendelian randomization (MR) may be a feasible alternative when randomized controlled trials are unethical or impossible. In this framework, genetic variants that are strongly associated with the potential risk factor are used as instrumental variables to determine whether the risk factor is a cause of the disease. MR studies are less susceptible to confounding and reverse causality compared with traditional observational studies (1). The basis for these studies is the random distribution of genetic polymorphisms at meiosis, which may provide a random distribution of exposure variables on the condition that they are closely related to a genetic marker, an allele, or a single nucleotide polymorphism (SNP). However, MR studies on the effect of alcohol on health are hampered by the lack of specific polymorphisms specifically addressing alcohol use. Nevertheless, the following covers first updated reviews and meta-analyses on alcohol and health since 2012 and thereafter the relevant MR studies.

## Physiology

### Nutritional aspects

Alcohol (ethanol) is generally consumed as beer (about 2.5–6 vol% alcohol), wine (about 12 vol%), or spirits (about 40 vol%). The energy liberated upon oxidation of alcohol in the body corresponds to 29 kJ per gram. At high alcohol consumption, however, the energy efficiency appears to be lower with relatively higher heat dissipation than with the other energy-yielding nutrients (3). Alcohol is efficiently absorbed through passive diffusion, mainly in the small intestine, and is distributed throughout the total water compartment of the body. Most of the absorbed alcohol is oxidized in the body but a small amount (5–10%) is lost through expired air and in the urine.

Replacing part of the food intake with alcoholic beverages can impair the quality of the diet. In particular, the consumption of dairy products, fruits, and vegetables appears to decrease when the intake of alcohol is increased. Some exceptions to this pattern, however, are noted. For example, a Danish study showed a strong positive association between fruit and vegetable consumption and wine intake, a finding that was supported by a systematic review of observational and ecological studies (4,5). Still, such associations may be biased by insufficient adjustment for social status and education and unknown residual confounding.

A high level of alcohol consumption can also result in impaired absorption of nutrients and increased nutrient loss in the urine. It also affects the nutritional status and disrupts the concentrations of trace elements, increasing the risk of enhanced oxidative stress and alcohol-related liver diseases (3, 6).

From a nutritional point of view, therefore, it is reasonable to recommend moderation in alcohol intake.

## Methods

The review follows the protocol developed within the NNR 2023 project (2). This scoping review is focused on areas with new scientific knowledge since the previous NNR, NNR2012 (7), that have special relevance for the Nordic and Baltic setting. In this review, however, the initial search string has been modified (e.g. by including ‘systematic reviews’, ‘meta-analysis’, ‘Mendelian randomization studies’ and other types of relevant literature) when appropriate, including *de novo* NNR systematic reviews.

One qualified systematic review (qSR) was identified in (8) for this review, namely *Alcohol consumption and all-cause mortality,* from the 2020 US Dietary Guidelines Advisory Committee (9).

The main literature search for this review was finalized on 31 May 2021 in MEDLINE with a search string: (“Alcohol Drinking”[Mesh]) AND (“Alcohol Drinking/epidemiology”[Mesh] OR “Alcohol Drinking/mortality”[Mesh]) covering the period 01 January 2012 to 31 May 2021.The full string is in the end of this paper. The search for systematic reviews and reviews resulted in 1,235 hits. Further search also included meta-analyses, MR studies, and relevant epidemiological observational studies, covering articles published between 1st January 2012 and 31st December 2020, with a complementary search up to 31st May 2021. The sources of evidence followed the eligibility criteria that were described previously (10).

Based on titles, 91 articles were identified, of which 27 were classified as reviews where prospective studies and randomized controlled trials were of primary interest, and 13 covered MR analyses considered relevant based on the full article. Additional 51 papers on background information were found via ‘snowballing’/citation chasing.

The search covered, as in NNR2012, the following topics: Cardiovascular diseases (CVD) and related risk factors, cancer, all-cause mortality (ACM), weight maintenance, prenatal exposure, and lactation. CVD were further subdivided into coronary heart disease, arrhythmias including atrial fibrillation, stroke (thrombosis, hemorrhages), congestive heart failure, cardio-metabolic risk factors (lipids, blood pressure, insulin and blood glucose, diabetes).

Inclusion and exclusion criteria were used based on relevance and importance in relation to FBDGs. Systematic reviews of systematic reviews were also included and checked regarding their references.

For this chapter, the articles were quality checked by using the tool AMSTAR 2 (11).

## Alcohol as a part of the dietary intake in Nordic and Baltic countries

Alcohol intake can be measured in many ways, and it is important to have in mind for which purpose these data are collected. One purpose is to include the alcohol intake in dietary assessment that enables description of alcohol intake as a percentage of the total energy intake in the given population. Such descriptions are given in Table 1, which shows that alcohol constitutes a higher percentage of the total energy intake among men than among women, and that Denmark has the highest intake of alcohol among a number of countries compared (12). Comparisons of these data to *sales* data from different countries data from WHO/the World Bank (13), however, show that the citizens of Latvia and Lithuania buy around 13 L of pure alcohol per adult, while Finland (11 L) and Denmark (10 L) have slightly lower figures, followed by Estonia, Sweden, and Iceland, all 9 L, and finally Norway (7 L).

**Table 1:** The dietary intake of alcohol as a percentage of total energy intake in the Nordic and Baltic countries

Country	Men (%)	Women (%)
Denmark	5.3	3.9
Finland	2.4	1.1
Iceland	2.3	1.5
Norway	2.5	2.1
Sweden	3.9	2.8
Estonia	3.2	0.7
Latvia	No data	No data
Lithuania	No data	No data

In the Nordic countries, mean alcohol consumption accounts for about 2 to 6% of the total energy intake in adults, but the intake is very unevenly distributed (14).

Several biomarkers of a high alcohol intake have been tested. The most used one during the last 25 years is the carbohydrate-deficient transferrin (CDT) level in blood, which for a long time has been used as biomarker of chronic alcohol abuse. Hence, while being an established indicator when monitoring alcohol intake among alcohol abusers, CDT has not proven valid as an indicator of alcohol intake in the *general* population (15). Several researchers have tried to develop other indicators, for instance, 5-hydroxytryptophol:5-hydroxyindole-3-atetic acid as a biomarker of recent alcohol intake (16), but none have proven useful in a public health setting. In population studies, alcohol intake is often self-reported data from longer lifestyle-related questionnaires.

It is important to note that when using this self-reported intake, the investigator is usually not interested in what amount has been consumed the last 24 h, but rather in obtaining a measure of the average intake over a longer period, as such information is usually more interesting to study as a predictor of the different health outcomes. It is generally assumed that alcohol intake often is misclassified or at least underreported, due to the taboo in the general population, when it comes to a high alcohol intake.

A way to validate the self-reported alcohol intake in a general population is to look at subsequent development of alcohol-specific disorders, such as alcoholic cirrhosis. Becker et al. studied this in a population-based prospective cohort of 13,285 men and women aged 30 to 79 years (17). They showed that women had a significantly higher relative risk of developing alcohol-related liver disease than men for any given level of alcohol intake, and that self-reported current alcohol intake was a good predictor of the future risk of alcohol-induced liver disease. It is, however, important to note that if these participants underreported their alcohol consumption, the true risk function would have been lower.

## Health outcomes relevant for Nordic and Baltic countries

### Alcohol and health

Alcohol is a toxic substance that affects all organs of the body. Both acute and chronic alcohol-induced damage contributes significantly to morbidity and mortality. The average alcohol consumption is a main determinant of the alcohol-related harm rates in a population, and the population tends to move in concert up and down the scale of consumption (18). There are exceptions from this pattern of collectivity, as shown in Sweden for the period 1990–2017, where an increase in per capita consumption was associated with a larger increase in the number of alcohol-related deaths among the lower than among more highly educated groups (19). The magnitude of the associations between average volume and heavy episodic drinking differed somewhat in strength across educational groups. Even so, they were clearly in the same positive direction. The authors conclude that their findings correspond to the notion of soft, rather than hard, collectivity, as discussed by Holmes et al. (20). Similar observations were seen in Finland, where a 33% reduction of alcohol excise taxes in 2004 resulted in increased alcohol-related mortality mainly among less privileged groups (unemployed and early-age pensioners) (21). The negative health effects of alcohol are primarily determined by the total amount of alcohol to which the body is exposed. This means that alcohol damage might develop in individuals who have not been visibly drunk. Daily consumption from five drinks and upwards per day is most likely to result in alcohol-related liver damage (22).

### Cardiovascular disease

Alcohol and its causal contribution to atherosclerotic disorders have been discussed for more than a century. In a paper from 1904, Richard C Cabot stated that most textbooks asserted the importance of alcohol in the production of arteriosclerosis, but he showed from post-mortem examinations that arteriosclerosis was a relatively rare condition in alcoholics (23). Alcohol has since then been extensively studied, displaying associations with coronary heart disease, atrial fibrillation, ischemic stroke, hemorrhagic stroke, and congestive heart failure, igniting vivid discussions on methodological issues associated with observational studies and their validity when assessing the effect of alcohol beverages.

#### Coronary heart disease

The main conclusions on alcohol and CVD in the NNR2012 were based on the meta-analysis by Ronksley et al. who published their meta-analysis in 2011 (24). They found that the pooled, adjusted relative risks (RR) for alcohol drinkers relative to nondrinkers were 0.71 (95% CI: 0.66–0.77) for incident coronary heart disease (CHD) (29 studies) and 0.75 (95% CI: 0.68–0.81) for CHD mortality (31 studies). The results persisted after excluding former drinkers from the category of abstainers. They concluded that the lowest risk of CHD occurred with 1–2 drinks a day, but for stroke at ≤1 drink per day. They also showed that mortality from all causes was reduced by 13% in drinkers compared with nondrinkers.

There are four additional meta-analyses with relevance to alcohol and CVD published after the NNR2012 (25–28). They differ slightly concerning study designs and population characteristics. Roerecke and Rehm included, for instance, both case–control and cohort studies (44 studies) in their analysis of chronic heavy drinking (on average ≥60 g pure alcohol/day) on the risk of ischemic heart disease (IHD) and covered about the same period as Ronksley et al. (25). They found that the pooled RR for IHD incidence, including both fatal and nonfatal events among chronic heavy drinkers was 1.04 (95% CI: 0.83–1.31) compared to lifetime abstainers. They concluded that there was no systematic evidence for a protective association from any type of chronic heavy drinking on IHD risk.

Wood et al. assessed the risk thresholds for alcohol consumption based on data from 599,912 current drinkers in 83 prospective studies (26). They showed a positive and curvilinear association between alcohol and total mortality, with the lowest risk for subjects drinking up to 100 g alcohol per week or 12.5 standard drinks. This was mainly because of a lower risk of MI. Any intake above that was associated with lower life expectancy. All other cardiovascular disorders in this database were positively associated with increased alcohol consumption. The association with MI was stronger for fatal than nonfatal cases. It is important to underline that Wood et al. studied current drinkers and did not include total lifetime abstainers.

Yoon et al. assessed seven studies on alcohol and CVD, the latter defined as CHD or stroke, both hemorrhagic and ischemic (27). They found a J-shaped association for men with no set point for when the CVD risk increased. Alcohol consumption level was classified into nonconsumers, light (0.01–10.0 g/day), light to moderate (10.1–20.0 g/day), moderate (20.1–40.0 g/day), moderate to high (40.1–60.0 g/day), and high (> 60.0 g/day). Light to moderate and moderate showed CVD incidence with RR of 0.68 (95% CI:0.57–0.81) and 0.72 (95% CI:0.58–0.90), respectively, compared with nondrinkers. There were no protective effects of light to moderate and moderate consumption on CVD incidence in men with 3–4 comorbidities including diabetes, hypertension, and dyslipidemia or those aged 40 or younger.

The review by Huang et al. included nine studies (11 cohorts) that focused on specific effects of alcohol consumption among hypertensive subjects (28). They assessed the effect of alcohol on CVD comprising both fatal and non-fatal MI, heart failure and stroke and ACM or total mortality in hypertensive subjects. Compared with the lowest alcohol level (abstainers/occasional drinkers), the pooled RR of CVD was 0.72 (95% CI:0.68–0.77) for the next category (median, 10 g/day), 0.81 (95% CI:0.71–0.93) for the second-highest category (median, 20 g/day), and 0.60 (95% CI:0.54–0.67) for the highest category (median, 30 g/day). There was a J-shaped relationship between alcohol use and total mortality, with the lowest risk estimate (RR: 0.82, 95% CI:0.76–0.88) at 8–10 g of alcohol consumption per day. They concluded that low-to-moderate alcohol consumption, here measured as 10–30 g alcohol/day, was inversely and significantly associated with the risk of CVD, whereas the lowest total mortality in individuals with hypertension was seen at 8–10 g alcohol/day.

The four additional meta-analyses added further support for a protective effect of alcohol on the risk of MI (25–28). The findings are pointing at a potential causal protective association between light to moderate alcohol intake and coronary artery disease, but the pattern is influenced by socio-economic status, suggesting unmeasured confounding influence.

We have identified the seven MR studies where alcohol was the causal factor of interest, with cardiovascular disease or associated risk factors as the main outcome (29–35). These studies found no protective effect of alcohol on the risk of coronary heart disease (29, 30, 35), whereas one reported a detrimental effect on peripheral arterial disease (31). The other studies do support an incremental effect of alcohol on systolic blood pressure, LDL-cholesterol, log-transformed triglycerides, and log-transformed fasting glucose.

The study by Millwood et al. is of particular interest as they assessed the association between alcohol and CVD, including CHD, both by a conventional cohort analysis and an MR instrument (35). The conventional analyses showed that self-reported alcohol intake had U-shaped associations, as the incidence of acute MI in men who reported drinking about 100 g of alcohol per week (1–2 drinks per day) had lower risks than nondrinkers or heavier drinkers. This was in contrast to genotype-predicted mean male alcohol intake, which did not have any U-shaped associations with risk.

The discrepant results between these epidemiological research avenues (conventional observational studies and MR) remain unexplained. A major point to be discussed is to what extent genetically determined exposure captures alcohol intake at the lower levels.

At the present state of knowledge, and based mainly on the observational conventional studies, we conclude that a drinking pattern in line with the previous NNR recommendations (<10 g/day for women and <20 g/day for men) is not detrimental concerning MI. The question of an upper protective limit for MI is still unsolved, but a daily intake of ≥60 g pure alcohol/day is associated with a substantially increased risk of cardiovascular disease.

#### Atrial fibrillation

Data from Framingham published almost two decades ago indicated little association between long-term moderate alcohol consumption and the risk of AF, but a significantly increased risk of AF among subjects consuming >36 g/day (approximatively >3 drinks/day) (36).

Larsson et al. assessed this association in a Swedish setting where they followed 79,019 men and women, accruing 859,420 person-years and identified 7,245 incident atrial fibrillation cases (37). Compared with current drinkers of <1 drink/week (12 g alcohol/drink), the RRs of AF were 1.01 (95% CI: 0.94–1.09) for <1 to 1 drinks/day, 1.07 (95% CI: 0.98–1.17) for 1 to 2 drinks/day, 1.14 (95% CI: 1.01–1.28) for >2 to 3 drinks/day, and 1.39 (95% CI: 1.22–1.58) for >3 drinks/day.

They also reported the results of a meta-analysis of seven prospective studies, including 12,554 AF cases, and showed that the RRs were 1.08 (95% CI: 1.06–1.10) for 1 drink/day, 1.17 (95% CI: 1.13–1.21) for 2 drinks/day, 1.26 (95% CI: 1.19–1.33) for 3 drinks/day, 1.36 (95% CI: 1.27–1.46) for 4 drinks/day, and 1.47 (95% CI: 1.34–1.61) for 5 drinks/day, compared with nondrinkers.

This corresponds to the results from HUNT, a population-based cohort study in Norway, comprising 47,002 participants where 1,697 validated AF diagnoses were registered during the 8 years of follow-up (38). The average alcohol intake was 3.8±4.8 g/day. The adjusted hazard ratio for AF was 1.38 (95% CI:1.06–1.80) for participants consuming >7 drinks per week compared with abstainers. There was virtually no association at <1 drink per day for women or at <2 drinks per day for men in the absence of binge or risky drinking. Risky drinking was defined as >7 drinks per week and >14 drinks per week for women and men, respectively. The population attributable risk among nonrisky drinkers was 0.07% (95% CI: –0.01 to 0.13%). Although alcohol consumption was associated with a curvilinear increasing risk of AF in general, the attributable risk of alcohol consumption within recommended limits among participants without binge or problem drinking was negligible in this population.

A review by Gallagher et al. (39) that encompassed seven studies, including Larsson et al. (37), reported that high, moderate, and low alcohol intake (defined by 3+, 1–2, <1 standard drink per day) was associated with the following hazard ratios (HR) in males 1.68, (95% CI: 1.18–2.41), 1.26, (95% CI: 1.04–1.54), 1.01, (95% CI: 0.82–1.24), and 1.29, (95% CI: 1.01–1.65), 1.03, (95% CI: 0.86–1.25), and 0.93, (95% CI: 0.82–1.05), in females. They conclude that low alcohol intake (up to 1 drink/day) was not associated with atrial fibrillation.

There are so far two MR studies that have assessed the association between alcohol and atrial fibrillation (40, 41). Lu et al. found that genetically predicted heavy alcohol consumption, defined as >35 /units/week for women and >50 units/week for men, increased the risk of AF independent of smoking, with OR 1.11 (95% CI: 1.06–1.16).

Jiang et al. performed a two-sample MR analysis to estimate the causal effect of alcohol consumption, alcohol dependence, and alcohol use disorder identification scores (AUDIT) on AF (32). The MR analyses revealed nonsignificant associations with alcohol consumption (odds ratio [OR] 1.004 [95% CI:0.796–1.266], alcohol dependence OR 1.012 [95%CI:0.978–1.048], and AUDIT score OR 0.889 [95%CI:0.433–1.822]). They conclude that their study did not support a causal effect of alcohol intake on the risk of AF.

In summary, the conventional observation studies indicate that alcohol consumption is a risk factor for atrial fibrillation, with increasing risk from drinking 2–3 standard drinks per day.

The conclusion on alcohol and AF in the NNR2012 was based on a review by Kodama et al. (42) and reads as follows ‘Results of the meta-analysis indicate that the risk of AF is probably increased by heavy drinking, while the effect of light to moderate intake is more uncertain due to a lack of high-quality studies’(7). The additional meta-analyses quoted above have clarified the picture, and it seems reasonable to conclude that light to moderate alcohol consumption (1–2 drinks/day) carries negligible risks of atrial fibrillation, whereas consumption above that level is consistently associated with increased risk. The differing results from the two MR studies only underline that further studies are needed, in particular a sufficiently large interventional study (40, 41).

#### Stroke

The NNR2012 for alcohol intake and stroke was based on two meta-analyses, one including a review published in 2011 (24, 43). Since then, one meta-analysis and two MR analyses have been published on the association between alcohol and stroke (31, 35, 44). Larsson et al. included data from 27 prospective studies on ischemic stroke (25 studies), intracerebral hemorrhage (11 studies), and/or subarachnoid hemorrhage (11 studies). Light and moderate alcohol consumption was associated with a lower risk of ischemic stroke, whereas more than 2–4 drinks a day was associated with an increased risk. The overall RRs were 0.90 (95% CI: 0.85–0.95) for less than 1 drink/day, 0.92 (95% CI: 0.87–0.97) for 12 drinks/day, 1.08 (95 % CI: 1.01–1.15) for more than 2–4 drinks/day, and 1.14 (95% CI: 1.02–1.28) for more than 4 drinks/day. Light and moderate alcohol drinking was not associated with any hemorrhagic stroke subtype. High alcohol consumption (>2–4 drinks/day) was associated with a nonsignificant increased risk of both hemorrhagic stroke subtypes, and the relative risk for heavy drinking (>4 drinks/day) was 1.67 (95% CI: 1.25–2.23) for intracerebral hemorrhage and 1.82 (95% CI: 1.18–2.82) for subarachnoid hemorrhage. Thus, less than 1 drink/day alcohol up to 1–2 drinks/day was inversely associated only with ischemic stroke, whereas >4 drinks/day was associated with increased risk of all stroke types with a stronger association for hemorrhagic strokes.

The findings from the observational studies are in contrast to those obtained from MR analyses. The MR study conducted in a Chinese population showed that increased alcohol consumption was associated with a higher risk of ischemic stroke and intracerebral hemorrhage but not with CHD (35). Millwood et al. obtained the RR estimates both by using conventional epidemiology (self-reported alcohol intake) and with genotype-predicted mean male alcohol intake. They showed that self-reported alcohol intake was U-shaped with the incidence of ischemic stroke, intracerebral hemorrhage, and acute MI, and that men who reported drinking about 100 g of alcohol per week (one to two drinks per day) had lower risks of all three diseases than nondrinkers or heavier drinkers. This was in contrast to the genotype-predicted mean male alcohol intake, which had a continuously positive log-linear association with stroke risk, but stronger for intracerebral hemorrhage (relative risk [RR] per 280 g per week 1·58, 95% CI: 1.36–1.84), than for ischemic stroke (1.27, 1.13–1.43).

Similar results were obtained by Larsson et al. who used MR to predict the effect of alcohol consumption on eight CVD, including stroke, CHD, AF, heart failure, venous thromboembolism, peripheral artery disease, aortic valve stenosis and abdominal aortic aneurism. |Up to 94 single-nucleotide polymorphisms were used as instrumental variables for alcohol consumption. Genetic association estimates for CVD were obtained from large-scale consortia and UK Biobank. They found that genetically predicted alcohol consumption was consistently associated with stroke and peripheral artery disease across the different analyses. The odds ratio (OR) per 1-SD increase of log-transformed alcoholic drinks per week was 1.27 (95% CI: 1.12–1.45).

Taken together, the results from the MR studies indicate that there is no protective effect of alcohol on the risk of stroke, neither ischemic nor hemorrhagic. The recommendations from NNR2012 should be revised accordingly.

#### Congestive heart failure

NNR2012 referred to a meta-analysis and review from 2010, which included six prospective cohort studies with a total of 164,479 study participants as a basis for their recommendations (45). They concluded that light to moderate drinking was not associated with increased congestive heart failure (CHF) risk and was associated with a lower risk of CHF. This was supported by a more recent meta-analysis by Larsson et al. (46).

These conclusions are seemingly in contrast to Wood et al. who among more than half a million alcohol consumers could not show any preventive effect of alcohol on the risk of heart failure (26). An MR study by van Oort et al. did not find any association between genetically determined alcohol consumption and heart failure (47). We conclude that alcohol is neutral concerning the risk of heart failure.

#### Cardio-metabolic risk markers

*Serum lipids* NNR2012 concluded that the favorable changes in certain cardiovascular biomarkers, and especially high-density lipoprotein (HDL), with increasing alcohol intake provided indirect physiological support for a protective effect of moderate alcohol use against CHD (48). Later studies have thrown doubts upon the causal role of HDL cholesterol as a cardioprotective factor. Holmes et al. concluded that there is a causal association between alcohol intake and triglyceride increase, whereas a direct effect on HDL cholesterol remains less certain (34). This was supported by Cho et al. (49). The uncertainty of HDL cholesterol as a plausible mechanism for the cardioprotective effect of alcohol has been strengthened by several observational longitudinal studies, here exemplified by an extensive Norwegian follow-up that showed that HDL cholesterol and self-reported alcohol intake were both, but independently, associated with CHD (50). The conclusion is that the HDL cholesterol coronary heart relationship most likely represents a noncausal association (51).

*Hypertension* There is convincing evidence that high alcohol intake is associated with increased blood pressure and risk of hypertension (48, 52), even if a significant protective effect has been reported for consumption at or below about 5 g per day in women (53). MR studies have on the other hand confirmed a positive effect of alcohol both on diastolic and systolic blood pressure (29–32). An MR study on the effect of alcohol on cardiovascular disease risk among diabetics and nondiabetic control subjects showed that low alcohol intake was associated with reduced systolic and diastolic blood pressure in men (33). The results suggest causal associations of alcohol consumption with blood pressure. There are only a few randomized controlled studies on the effect of decreasing alcohol intake on systolic and diastolic blood pressure and subsequent morbidity and mortality (54). Such studies are needed to provide additional evidence on this specific question.

*Insulin and glucose concentrations* Reviews and meta-analyses are sparse regarding insulin and blood glucose, but individual studies have found that an alcohol intake of 1–2 drinks per day is associated with reduced fasting insulin concentration and improved insulin sensitivity (55–59). Furthermore, fasting glucose levels were similar in nondrinkers and moderate alcohol drinkers in a prospective cohort study (60). More studies are needed to further clarify this question.

*Diabetes* Two systematic reviews on the association between alcohol consumption and diabetes were identified (61, 62). Knott et al. based their analysis on 38 studies representing more than 1.9 million participants and 125,926 cases of type 2 diabetes. Reductions of type 2 diabetes risk were present at all levels of alcohol intake <63 g/day, with risks increasing above this threshold. This finding was based on using all abstainers, disregarding reason for abstention, as reference group. The largest risk reduction was observed at intake of 1–14 g alcohol a day. The authors conclude that reductions in risk might be confined to women and non-Asian populations. They also underlined that the magnitude of the risk reduction may have been overestimated using a referent group contaminated by less healthy former drinkers (61). In a later work, Li et al examined 26 studies with 31,621 cases and more than 700,000 individuals. They concluded that alcohol consumption of <30 g/day seemed to be associated with a lower risk of T2D in the whole population. Similarly, alcohol consumption of <20 g/day seemed to be related to a lower risk of T2D in women, and consumption <40 g alcohol/day was associated with a lower risk of T2D in men. Higher alcohol consumption was not related to this risk (62).

A meta-analysis covering prospective cohort studies from January 1966 to February 2016 assessed the effects of specific types of alcoholic beverages on the risk of type 2 diabetes (63). Wine consumption was associated with a significant reduction of the risk of type 2 diabetes, with the pooled RR of 0.85, whereas beer or spirits consumption led to a slight trend of decreasing risk of type 2 diabetes (relative risk 0.96, 0.95, respectively). The dose–response analysis showed a U-shaped relationship for each of the three alcohol types and type 2 diabetes. The peak risk reduction emerged at 20–30 g/day for wine and beer, respectively, and at 7–15 g/day for spirits, with a decrease of 20, 9 and 5%. The authors suggest that wine might be more helpful for protection against type 2 diabetes than beer or spirit.

The above findings from observational studies deviate from an MR study in China by Peng et al. They concluded that higher alcohol intake appears to be causally associated with increased diabetes risk and worsened related traits, even for moderate drinkers, without any U-related or curvilinear association (64). Even if curvilinearity should exist, they argue that it may be too weak to be detected, which indicates that the benefit of moderate alcohol consumption is limited and has little implication for clinical practice.

Based on the discrepant results between conventional observational studies and genetical instrumental analyses, we find it premature to draw any firm conclusions on the association between alcohol consumption and diabetes.

*Subclinical atherosclerosis* There are no reviews published on the association between alcohol consumption and subclinical atherosclerosis, but data are available from a European multicenter study where one assessed subclinical atherosclerosis by measuring carotid intima-media thickness (C-IMT) and its 30-month progression in 1,772 men and 1,931 women aged 54–79 years with high risk for CVD (65).

Self-reported alcohol consumption was categorized as follows: none (0 g/day), very-low (0 to 5 g/day), low (> 5 to ≤ 10 g/day), moderate (> 10 to ≤ 20 g/day for women, > 10 to ≤ 30 g/day for men) and high (> 20 g/day for women, > 30 g/day for men). The differences between median C-IMT values in different levels of alcohol consumption (vs. very low) showed that moderate alcohol consumption was associated with lower C-IMT at baseline and with lower C-IMT after 30 months when adjusted for sex, age, physical activity, education, smoking, diet and latitude. The authors concluded that there was an inverse relation between moderate alcohol consumption and carotid subclinical atherosclerosis and its 30-month progression, independently of several potential confounders.

There is at the present insufficient data to provide any recommendations concerning alcohol consumption and subclinical atherosclerosis.

### Cancer

Alcohol (ethanol) has for decades been classified as a human carcinogen by the International Agency for Cancer Research, and it has recently been shown, that 4.1% of all cancers are alcohol-related (66). The 2020 World Cancer Report (WCRF) includes an extensive systematic review of the available evidence on the association between alcohol intake and the development of cancer (67). Evidence was graded as ‘convincing’ for an increased risk of cancer of *the mouth, pharynx, larynx, esophagus, liver, and colorectal cancer among men and breast cancer among women*. There was ‘probable’ evidence for an association between alcohol intake and the risk of liver cancer and colorectal cancer among women. Several subsequent cohort studies, meta-analyses, and reviews have been published (68–70). For the cancers with sufficient evidence in the WCRF report, new studies have supported the evidence of a relation between alcohol intake and cancer risk (71–76). This is especially the case for cancers of the upper aerodigestive tract and colorectal cancer in both sexes and breast cancer among women (77, 78).

A recent large cohort study showed a significant association between heavy alcohol intake and increased risk of pancreatic cancer (79). There was no association between moderate drinking and pancreatic cancer risk. However, because smoking is a strong risk factor for pancreatic cancer, residual confounding is a potential problem in these studies (80). Residual confounding in this case would imply that smoking is imperfectly measured and adjusting for smoking will not completely remove this effect.

This could also be the case in the studies between alcohol intake and lung cancer, where a suggestive increased risk has been reported. Interestingly, an MR study on alcohol intake and lung cancer has found a protective effect, if taken with meals (81). The causal relationship analyses between habitual alcohol consumption, defined as < 30 g/day, with meals and some risk factors for cancers showed that this alcohol consumption habit was a beneficial factor for reducing body mass index and the number of cigarettes smoked per day. No strong association was shown between alcohol intake and the risk of ovarian, endometrial, or non-Hodgkin lymphoma (82, 83).

A review on alcohol consumption and prostate cancer (84) concluded that daily consumption of up to about three drinks per day does not appear to influence prostate cancer risk, whereas heavy consumption of seven or more drinks per day might be associated with an increased risk. However, a more recent meta-analysis has suggested that alcohol may be a risk factor for prostate cancer (85).

The overall conclusion is that the evidence for associations between alcohol intake and cancer does not show any ‘safe limit’ of intake. This is especially true for breast cancer where even very moderate intake has been shown to increase the risk (67). Although a few studies have suggested differently, the effect from ethanol seems irrespective of the type of drink, but this may be difficult to disentangle due to the heterogeneity across the studies included in the meta-analyses (68).

## All-cause mortality

All-cause mortality is, as indicated by the name, constituted by many different causes of death. The risk for some of these, such as several cancers, cirrhosis, drug abuse, accidents, and violent deaths, is increased with increased intake of alcohol. For other causes, such as cardiovascular disease and type 2 diabetes, the risk has often been shown to be reduced at the lower levels of light alcohol intake, resulting in a so-called J-shaped relation between alcohol and ACM.

During the last 10 years, three meta-analyses of the relation between alcohol and mortality have appeared (86–88). A systematic review including a meta-analysis by Stockwell et al., based on 87 prospective cohort studies, included a series of sub-analyses of inclusion of ex-drinkers in reference category and effect modification by different factors (88). Unadjusted analyses showed a J-shaped relation between alcohol and mortality with a reduced risk (RR=0.85 (95% CI: 0.83; 0.90) among subjects with low consumption (<25 g alcohol/day) and increased risk (RR=1.29 (95% CI: 1.22; 1.36) among heavy drinkers (≥65 g alcohol/day), both compared to nondrinkers.

Adjustment for abstainer biases and quality-related study characteristics, however, changed the effect estimates to no significant reduction in mortality risk for low-volume drinkers (RR = 0.97, 95% CI [0.88, 1.07]). They found no significant interaction with sex. Stockwell et al. concluded that adjusting for these factors shows that low-volume alcohol consumption has no net mortality benefit compared with lifetime abstention or occasional drinking.

The study by Wang et al. aimed at evaluating the effect of drinking on the risk of ACM in women compared with men (86). A J-shaped dose–response relationship was confirmed between alcohol and ACM both in men and women, but there was an increased risk for women compared to men at higher consumption. The female-to-male relative risk rates of ACM were 1.52 (95% CI: 1.01, 2.29), 1.95 (95% CI: 1.08, 3.49), and 2.36 (95% CI: 1.15, 4.88), respectively, for those who consumed 75, 90, and 100 g/day of alcohol. Compared with nondrinkers, the RR of ACM was 0.95 (95% CI: 0.92, 0.98), 0.92 (95% CI: 0.85, 0.99), 0.96 (95% CI: 0.83, 1.10), 1.15 (95% CI: 0.92, 1.43), 1.36 (95% CI: 1.02, 1.80), and 1.56 (95% CI: 1.12, 2.19), respectively, for men who consumed 10, 25, 50, 75, 90, and 100 g/day of alcohol. Corresponding figures for women were 0.93 (95% CI: 0.90, 0.96), 0.91 (95% CI: 0.85, 0.96), 1.09 (95% CI: 0.93, 1.27), 1.74 (95% CI: 1.23, 2.47), 2.65 (95% CI: 1.59, 4.42), and 3.70 (95% CI: 1.95, 7.04). Thus, there was no difference in risk among men and women in the lower range of alcohol intake, while there were significant sex differences in the higher intake categories (> 25 g/day).

Jayasekara et al. identified nine cohort studies published during 1991–2010 (comprising 62,950 participants and 10,490 deaths) (87). They confirmed that there was a lower mortality risk in men with low levels of alcohol intake over time but a higher mortality risk for those with intakes over 40 g/day compared with abstainers. The pooled RR was 0.90 (95% CI: 0.81, 0.99) for 1–29 g/day, 1.19 (95% CI: 0.89, 1.58) for 30–59 g/day, and 1.52 (95% CI: 0.78, 2.98) for 60 or more g/day compared with abstention. Jayasekara et al. did not include enough women to study any gender differences.

The incidence and relative distribution of alcohol-related diseases differs by age and so does the ACM. The magnitude (or the sheer existence) of a J-shaped association between alcohol and ACM will therefore be a reflection of the dominating causes of death in each age group. The nadir (representing the alcohol intake at the lowest risk of mortality) is achieved at a lower intake at younger ages. In a British study, the lowest mortality risk among men and women 16 to 34 years old was observed among the nondrinkers (89). Hence, a beneficial effect of alcohol is not observed among the young, and instead, alcohol is directly associated with increased ACM in this age group.

Results from studies regarding the role of drinking patterns consistently imply an increased mortality risk associated with drinking large amounts of alcohol per session or binge drinking (29). Furthermore, there is good evidence that the protective effect of alcohol on cardiovascular disease only occurs if the pattern of drinking is not a binging pattern (90). Hence, the J-shaped association between alcohol intake and ACM depends upon the drinking pattern.

The nadir of the ‘J’ reflects a relatively lower risk of CHD among light to moderate drinkers compared with abstainers and the ascending leg of the J is reflective of an increased risk of alcohol-related diseases such as liver cirrhosis, pancreatitis, upper gastrointestinal cancers, cardiomyopathy, polyneuropathy, and deaths from accidents and violence among excessive alcohol users. Because the association between alcohol and ACM represents the sum of the numerous diseases and outcomes that are related to alcohol, the shape and nadir of the risk curve depend on the distribution of other variables such as age, relative incidences of diseases, the prevalence of drunk-driving, etc. Thus, the association between alcohol and ACM does not have the same causal interpretation as associations between alcohol and singular endpoints.

In conclusion, light to moderate drinking is not associated with increased mortality risk, it is on the contrary likely to be associated with a lower risk among middle-aged and older adults who do not engage in episodes of heavy drinking. Total abstinence is associated with the lowest risk of mortality in young adults, and binge drinking should be avoided in all age groups.

## Weight maintenance

Results from reviewing 31 prospective cohort studies and clinical trials did not show any consistent associations between alcohol intake and weight gain. Some studies, however, found that higher levels of consumption (>2–3 drinks/day) were associated with weight gain. The type of beverage seems to be of importance with a lower weight gain and waist-to-hip ratio observed for wine compared to beer and spirits (91). Only four prospective studies reported on the relation between alcohol intake and waist circumference or waist-to-hip ratio. The findings were inconsistent with studies finding positive, negative, or no associations. The effect of alcohol on weight gain and waist circumference is not clear from the current evidence, and no conclusion could be drawn.

## Prenatal alcohol exposure

Alcohol can affect the developing fetus in a dose-dependent manner. Alcohol is teratogenic and can lead to fetal alcohol syndrome (FAS), which is characterized by craniocephal abnormalities, physical and mental retardation, and cardiac and joint abnormalities These effects are mainly seen with an alcohol intake above 24–48 g/day (92). Systematic reviews of prenatal alcohol exposure have found that low to moderate levels of alcohol consumption had no consistently significant effects on miscarriage, stillbirth, intrauterine growth restriction, prematurity, birth weight, small for gestational age at birth, or birth defects (93, 94).

However, since the limit between ‘light’ and ‘moderate’ intake among pregnant women is still weakly defined, and there are no health benefits, drinking in pregnancy should still be avoided.

## Alcohol intake during lactation

There are no indications of medical consequences in the child if a lactating mother occasionally drinks small amounts of alcohol (95–97). The amount of alcohol in nursing infants through breast milk is a very small percentage of the maternal dose, and the baby can metabolize this small dose. Binge drinking is of course another risk factor (for mistreating the infant etc.) and should be avoided.

## Requirement and recommended intakes

Alcohol consumption is associated with both negative and positive health effects. Current evidence from mainly conventional epidemiological studies suggests that regular, moderate alcohol consumption confers a modest protective effect against MI and possibly type 2 diabetes among middle-aged and older individuals, while alcohol consumption among young adults is detrimental, especially because of the tendency to binge drinking in this age group. For several cancers, there is convincing evidence that alcohol consumption increases the risk, and it is not possible to set any ‘safe limit’ of intake. This is especially true for breast cancer, where even moderate intake has been shown to increase the risk. Light to moderate regular alcohol consumption is not associated with increased mortality risk among middle-aged and older adults.

Based on the overall evidence, it is recommended to limit alcohol intake. Based on estimates of the maximal mortality risk reduction associated with moderate alcohol consumption, the intake should not exceed 20 g (approximately two units) per day for both women and men. The consumption of alcohol should not exceed 5% of the energy intake in adults. Pregnant women, children, and adolescents are recommended to abstain from alcohol. Lactating women are recommended to follow the above.

## Data gaps for future research

We have previously alluded to the pitfalls and potential sources of bias associated with observational epidemiological studies, especially the abstainer bias where previous alcohol consumers are lumped together with lifelong abstainers. The researchers have circumvented this problem by using the lowest exposure categories as the reference groups, but this does not fully ensure a comparison between non-exposure and exposure with regard to alcohol. Further refined methods for assessing the true alcohol intake in observational studies are warranted.

The need for experiments or trials with the effect of alcohol on harder endpoints is considered unethical and to a large extent unfeasible. There are obvious ethical issues, but trials assessing the effect of low doses of alcohol on mediators and risk factors such as blood glucose, blood pressure, blood lipids, and inflammatory markers should be encouraged together with gene–alcohol interaction studies.

The instrumental variable approach using MR is a promising alternative to randomized controlled trials, and further studies using this framework should be encouraged and especially the search for specific polymorphisms specifically addressing alcohol use.

## Conflict of interest and funding

Both authors declare that they have no conflicts of interest. The authors have not received any funding or benefits from industry or elsewhere to conduct this study.

## Search string

(Alcoholics[Majr] OR Alcoholic Beverages[Majr] OR Alcohol Drinking[Majr] OR “Alcohol-Related Disorders”[Majr:NoExp] OR “Alcohol-Induced Disorders”[Majr] OR “Alcohols/adverse effects”[Majr:NoExp] OR “Ethanol/adverse effects”[Majr:NoExp] OR “Alcohols/toxicity”[Majr:NoExp] OR “Ethanol/toxicity”[Majr:NoExp] OR ((alcohol[ti] OR alcoholism[ti] OR alcoholics[ti] OR (alcoholic[ti] NOT non-alcoholic[ti]) OR (drink*[ti] NOT water[ti]) OR drunk*[ti] OR beer*[ti] OR wine[ti] OR wines[ti] OR (ethanol[ti] AND (consum*[ti] OR drink*[ti] OR drunk*[ti] OR intake[ti] OR intox*[ti] OR abus*[ti] OR misuse[ti] OR mis-use[ti]))) NOT medline[sb])) AND (“Systematic Review” [Publication Type] OR “Meta-Analysis” [Publication Type] OR metaanalysis[Title] OR metaanalyses[Title] OR “meta-analysis”[Title] OR “meta-analyses”[Title] OR (systematic[Title] AND review[Title]) OR “synthesis review”[Title] OR metasynthesis[Title] OR metasyntheses[Title] OR “meta-synthesis”[Title] OR “meta-syntheses”[Title] OR metaregression[Title] OR “meta-regression”[Title] OR “synthesis of evidence”[Title] OR “evidence synthesis”[Title] OR “evidence syntheses”[Title] OR “evidence-based synthesis”[Title] OR “scoping review”[Title] OR “umbrella review”[Title] OR ((review[Title] OR overview[Title] OR summary[Title] OR synthesis[Title] OR syntheses[Title]) AND “systematic reviews”[Title]) OR “Mendelian Randomization Analysis”[Mesh] OR “Mendelian Randomization”[Title]) AND (English[lang] OR Norwegian[lang] OR Swedish[lang] OR Danish[lang]) AND “last 10 years”[PDat]))) NOT (“pancreatitis 1”[All Fields])) NOT (“smoking”[All Fields])) NOT (“psychiatry”[All Fields])) ) NOT (“multiple sclerosis”[All Fields])
